# Climb’s black and ethnic minority information project (BEMIS)

**DOI:** 10.1186/1750-1172-7-S2-A36

**Published:** 2012-11-22

**Authors:** Steve Hannigan

**Affiliations:** 1 CLIMB, 176 Nantwich Road, Crewe, Cheshire CW2 6BG, UK

## 

Our unique three year project enabled us to film and catalogue a library of 110 DVDs covering many inherited metabolic diseases, diagnosis, symptoms, treatment, research and prognosis. This information was then transcribed into written format and translated into various languages.

This innovative project grew out of a need identified during a 2 year research project for information covering metabolic diseases. Our work with families from an ethnic minority background in the West Midlands of England showed that there was an identifiable gap in our service provision and in order to meet this need we would have to change the way we worked and the information that we provided to families, adults and professionals.

Our information needed to be clear, simple and easily accessible. Unfortunately, the rareness of these conditions meant that Climb’s existing information in English, was hard to access for many families, communities and people who did not read English as their first language. Following on from our research project and our work with minority communities and travellers, we identified diseases, languages and formats needed for our project. We chose 110 metabolic conditions and two languages to translate the information into. We also produced a variety of leaflets and posters to convey this information. Many specialists agreed to be filmed. The aim was to produce disease specific information on a DVD initially into two languages Urdu and Punjabi, which would cover many important aspects of inherited metabolic diseases including:

Symptoms - Long term effects - Treatment - Diagnosis - Dietary requirements.

Care - Long term treatment options - Specialist treatment - Research - Prognosis.

Inheritance factors - Genetics.

Initial translations and DVDs led to requests for more leaflets in more languages. Our final list of languages used was Arabic, English, Cantonese, French, Danish, Greek, Hebrew, Japanese, Persian, Portuguese, Punjabi, Russian, Spanish, Urdu, Welsh.

On occasion, Climb was able to meet service users’ requests to provide specific information on a particular aspect of Inherited Metabolic Diseases such as pregnancy.

Conclusion - Climb has created a free multi ethnic resource for anyone needing information about metabolic diseases which has been translated into several languages. In addition, two hospitals have received 7 DVDs for their resource library. By the end of the project we had sent out over DVDs.

A Project Funded by the UK Department of Health and Climb.

